# Reprogramming Primordial Germ Cells into Pluripotent Stem Cells

**DOI:** 10.1371/journal.pone.0003531

**Published:** 2008-10-27

**Authors:** Gabriela Durcova-Hills, Fuchou Tang, Gina Doody, Reuben Tooze, M. Azim Surani

**Affiliations:** 1 Wellcome Trust/Cancer Research UK Gurdon Institute of Cancer and Developmental Biology, University of Cambridge, Cambridge, United Kingdom; 2 Division of Experimental Haematology, Leeds Institute of Molecular Medicine, University of Leeds, Leeds, United Kingdom; Ecole Normale Supérieure de Lyon, France

## Abstract

**Background:**

Specification of primordial germ cells (PGCs) results in the conversion of pluripotent epiblast cells into monopotent germ cell lineage. Blimp1/Prmt5 complex plays a critical role in the specification and maintenance of the early germ cell lineage. However, PGCs can be induced to dedifferentiate back to a pluripotent state as embryonic germ (EG) cells when exposed to exogenous signaling molecules, FGF-2, LIF and SCF.

**Methodology and Principal Findings:**

Here we show that Trichostatin A (TSA), an inhibitor of histone deacetylases, is a highly potent agent that can replace FGF-2 to induce dedifferentiation of PGCs into EG cells. A key early event during dedifferentiation of PGCs in response to FGF-2 or TSA is the down-regulation of Blimp1, which reverses and apparently relieves the cell fate restriction imposed by it. Notably, the targets of Blimp1, which include c-Myc and Klf-4, which represent two of the key factors known to promote reprogramming of somatic cells to pluripotent state, are up-regulated. We also found early activation of the LIF/Stat-3 signaling pathway with the translocation of Stat-3 into the nucleus. By contrast, while Prmt5 is retained in EG cells, it translocates from the nucleus to the cytoplasm where it probably has an independent role in regulating pluripotency.

**Conclusions/Significance:**

We propose that dedifferentiation of PGCs into EG cells may provide significant mechanistic insights on early events associated with reprogramming of committed cells to a pluripotent state.

## Introduction

Primordial germ cells (PGCs) are the embryonic precursors of the germ cell lineage, which are restricted to form only sperm and eggs following their specification from pluripotent epiblast cells. Evidence suggests that Blimp1 is the key determinant of germ cell specification as it initiates the germ cell-specification program in pluripotent epiblast cells at embryonic day (E) E6.5–7.5 [1]. Furthermore, the Blimp1/Prmt5 complex plays a decisive role not only during specification of founder population of PGCs, but also thereafter in maintenance of early germ cells as they migrate into the developing gonads between embryonic day (E) 8.5–11.5 [2]. It is precisely during this interval that PGCs can be induced to dedifferentiate into pluripotent embryonic germ (EG) cells in vitro when exposed to exogenous signaling molecules, LIF, FGF-2 and SCF [3]–[5]. While PGCs show expression of some key pluripotency-specific genes, they differ significantly from EG cells in being monopotential, and unlike EG cells, they are incapable of participating in chimeras when introduced into blastocysts [5]. The process of dedifferentiation of PGCs into EG cells signifies the reversal of their developmental program, which may provide insights into how a phenotypically and developmentally restricted group of cells can undergo dedifferentiation into self-renewing pluripotent stem cells.

Cell culture induced reprogramming has been successfully achieved with PGCs isolated from E8.0–12.5 old embryos, but the efficiency of reprogramming declines particularly after E11.5 of development (unpublished observation), presumably because PGCs undergo major epigenetic and phenotypic changes upon entry into the developing gonads and they are no longer able to respond to the environmental cues [6]. Recently we have shown that the conversion of PGCs to EG cells takes approximately 10 days as judged by monitoring the ability of these cells to make chimeras [5]. Several mutations are known to increase the efficiency of generating embryonic germ (EG) cells, including *Dnd*, *Pten*, *Pgct1 and Akt* signaling [7]–[10]. However, so far the presence of cytokines, including FGF-2, LIF and SCF seems to be essential for this process. Notably, the presence of the added FGF-2 seems to be critical only for the first 24 hours as it is dispensable thereafter [5]. This transient ability to respond to FGF-2 indicates that some critical event(s) is triggered by it that is associated with the initiation of reprogramming of PGCs into EG cells. However, little is yet known the mechanism accompanying this process, which we are attempting to elucidate.

We set out to examine the critical steps that lead up to the conversion of PGCs to EG cells. Our work has identified important sequential steps during reprogramming of PGCs to pluripotency. We suggest that Blimp1 has an important role in preventing PGCs from dedifferentiation into pluripotent stem cells, and its down-regulation is an early key event, which leads to the up-regulation of some of its key targets, amongst which are c-Myc and Klf-4, while the activation of LIF/Stat-3 pathway is important for the self renewal of EG cells.

## Results

### Identification of differentially expressed genes between PGCs and EG cells

First we set out to identify some key differences between PGCs and EG cells, to discover important markers and potential regulators of dedifferentiation of PGCs into EG cells. We performed both representative differential analysis (RDA) screen and expression analyses of selected genes associated with pluripotency and/or self-renewal. RDA screen was performed between cDNA libraries made from E11.5 PGCs and EG cells and an expression analysis was performed using single cells complementary DNAs (sc cDNAs) made from PGCs isolated from E8.5 embryos, and EG cells.

The expression of 27 genes we examined in PGCs and EG cells is summarised in Table 1. Amongst these are 14 genes that are expressed in both the PGCs and EG cells. Some genes, including Oct-4 and Sox-2, were detected in cultured PGCs, and their expression continued at high levels throughout the 6-days of culture in FGF-2, LIF and SCF (Figure S1). The second group of 13 represents differentially expressed genes in PGCs and EG cells. Amongst these genes are c-Myc, Stat-3, Klf-4, Dnmt3L and Eras, which were detected predominantly, if not exclusively, in EG cells and not in PGCs. *Dnmt3L* was identified in the RDA screen as one of the genes that is expressed in EG cells but not in the PGCs. By contrast, Blimp1 is the key gene that is associated with PGCs and not detected in EG cells [2].

**Table 1:** Expression analysis of 27 genes.

Gene	8.5 dpc PGCs				EG cells			
	5	7	14	20	1	2	3	6
**Group I**								
Oct-4	+	+	+	+	+	+	+	+
Sox-2	+	+	+	+	+	+	+	+
nanog	+	+	+	+	+	+	+	+
Dppa5(Esg-1)	+	+	+	+	+	+	+	+
Sall4	+	+	+	+	+	+	+	+
mRif1	+	+	+	+	+	+	+	+
E-cadherin	+	+	+	+	+	+	+	+
TNAP	+	+	+	+	+	+	+	+
Rex1 (Zfp42)	+	+	+	+	+	+	+	+
Utf1	+	+	+	+	+	+	+	+
Dppa3 (Stella)	+	+	+	+	+	+	+	+
Sox15	+	+	+	+	+	+	+	+
Grb2	−	+	+	+	+	+	+	+
β-catenin	−	+	+	+	+	+	+	+
**Group II**								
***c-Myc***	**−**	**−**	**−**	**+**	**+**	**+**	**+**	**+**
***Stat-3***	**−**	**−**	**−**	**−**	**+**	**+**	**+**	**+**
***Klf-4***	**−**	**−**	**−**	**−**	**+**	**+**	**+**	**+**
Dnmt3L	−	−	−	−	+	+	+	+
ERas	−	−	−	−	+	+	+	+
Fthl-17	−	−	−	−	+	+	+	+
Dppa2	−	−	−	−	+	+	+	+
Dppa4	+	−	−	−	+	+	+	+
Ecat1	−	−	−	−	+	+	+	+
Ecat8	−	+	+	−	+	+	+	+
Gdf3	+	+	+	+	+	−	−	−
Fbxo15	−	+	−	+	+	+	+	+
Tcl1	−	+	−	−	+	+	+	+

Group I – genes in this group were expressed in both PGCs and EG cells.

Group II – genes were expressed differentially in PGCs and EG cells, and shown as as positive (+) and negative (−).

E8.5 PGCs samples tested for expression are referred to as, 5, 7, 14, 20.

EG cells tested for expression are referred to as 1, 2, 3, 6.

### Loss of lineage restriction in PGCs cultured in vitro

**Figure 1:**
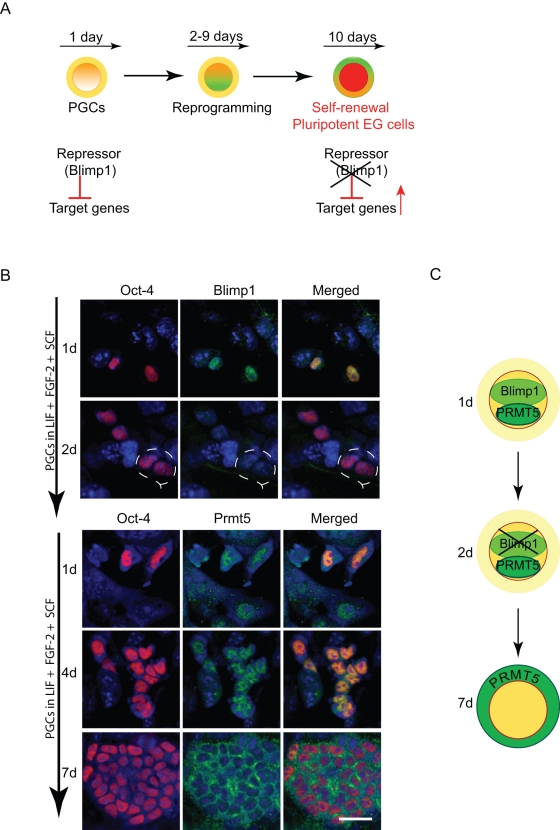
Expression of Blimp1 and Prmt5 during PGCs reprogramming to EG cells. (A) Schematic depicting dedifferentiation of PGCs to EG cells in the presence of LIF, FGF-2 and SCF. PGCs undergo reprogramming resulting in a large colony of pluripotent EG cells after 10 days of culture. Down-regulation of Blimp1 repressive complex may be a key early event leading to the generation of EG cells. (B) Expression of Blimp1 and Prmt5 in cultured PGCs. Oct4 is a marker of PGCs (red). Blimp1 (green) was detected on day 1 (labelled as 1d) of culture but not on 2d (dashed line). Prmt5 (green) was detected on day 1–4 in the nuclei of PGCs, but subsequently translocated to the cytoplasm on day 7 of culture. Merged images are shown with DNA stained with Toto-3 (blue). Scale bar, 30 µm. (C) The fate of Blimp1/Prmt5 complex during PGCs dedifferentiation to EG cells is depicted schematically.

First, we asked if there was any change in the expression of Blimp1 during the culture of PGCs under the conditions when they undergo reprogramming into EG cells. This is because expression of Blimp1 has been identified as the key event during the specification of PGCs from pluripotent epiblast cells [1]. Furthermore, the Blimp1/Prmt5 complex is probably important for maintaining the identity of the early germ cell lineage following the specification of PGCs, especially while germ cells migrate into the developing gonads between E8.5–E11.5 [2]. We have suggested previously that derivation of EG cells from PGCs may be associated with the loss of Blimp1, as it is likely to be crucial for ‘repressing’ or maintaining PGCs from acquiring an overtly pluripotent stem-cell phenotype [2]. We therefore anticipated that reprogramming of PGCs into EG cells may be coupled with down-regulation of Blimp1 and up-regulation of the targets of this repressor complex (Figure 1A).

Indeed, we found that Blimp1 (but not Prmt5- see below), which is expressed in PGCs but not in EG cells (Figure S2), is rapidly down regulated in PGCs when they are cultured in FGF-2, LIF and SCF. Whereas after day 1 of culture we could still detect Blimp1 in nuclei of all examined PGCs, this was not the case on day 2 of culture (Figure 1B). To determine if the early loss of Blimp1 expression is an important event during the conversion of PGCs to EG cells, we cultured PGCs in the absence of FGF-2, while LIF and SCF were still present. In this control experiment, PGCs fail to undergo reprogramming into EG cells [3]. Strikingly, we found that Blimp1 failed to be down-regulated under these conditions (data not shown). This suggests that down-regulation of Blimp1 is probably an important early event that is associated with reprogramming of PGCs. Notably, the early down-regulation of Blimp1 is also linked with the absolute necessity for the presence of FGF-2 in the medium for the first 24 hours, since adding this cytokine after this time does not result in the reprogramming of PGCs to EG cells [5]. It is not possible at present to ask if PGCs with a loss of Blimp1 can undergo reprogramming with or without the presence of FGF-2 since this results in aberrant PGC-like cells that fail to proliferate [1].

In contrast to Blimp1, we detected Prmt5 in nuclei of PGCs cultured for the first seven days but thereafter Prmt5 underwent nuclear-to-cytoplasmic translocation in large colonies of cells resembling EG cells (Figure 1B, Figure S2 B). However, Prmt5 continued to be seen in the nuclei of some small colonies of cells, indicating that these cells may not have converted to EG-like cells. Yet the loss of Blimp1 is probably the critical event. This is because Blimp1 has five zinc fingers, which would allow recruitment of the histone methyltransferase, Prmt5, to specific DNA target sequences by the Blimp1/Pmt5 complex. Prmt5 itself has no apparent specificity for binding to DNA. We have previously shown that the Blimp1/Prmt5 complex has the potential to repress target loci through symmetrical dimethylation of arginine 3 on histone H2A/H4 (H2A/H4R3me2s) [2].

### Up regulation of Blimp1 targets in cultured PGCs

If Blimp1/Prmt5 complex is involved in maintaining the germ cell phenotype, we would expect that some targets of the complex would be up-regulated, which are repressed during specification of PGCs, and during their subsequent migration into the developing gonads. One known target of the Blimp1/Prmt5 complex is *Dhx38*, which is not detected in migrating PGCs until E11.5, but it is detected at E12.5 when the Blimp1/Prmt5 complex translocates from the nucleus to cytoplasm [2]. While we did not detect Dhx38 during the first 4-days of PGCs in culture, we could detect it as a weak signal on day 7 of culture when we started to observe small EG-like colonies. However, in large multi-cellular EG colonies on day 8 of culture, Dhx38 was expressed at high levels (Figure S2 C), which coincides with delocalisation of Prmt5 from the nucleus to cytoplasm. These observations suggest that Blimp1/Prmt5 may maintain early germ cells through repression of target genes of which *Dhx38* is an example.

**Figure 2:**
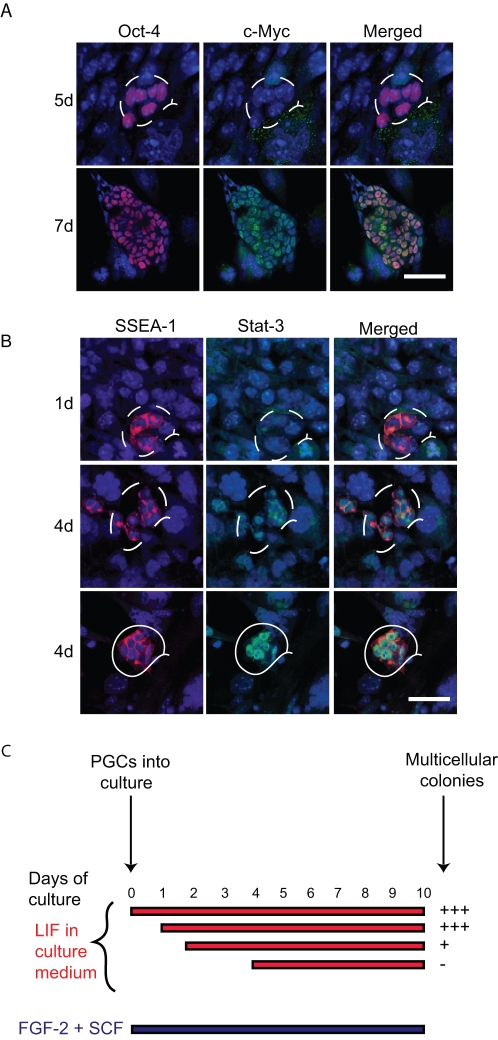
c-Myc and Stat-3 expression is up-regulated in cultured PGCs. PGCs were cultured in FGF-2, LIF and SCF. Oct-4 is a marker of PGCs (red). (A) Immunofluorescence staining of c-Myc (green) showed no expression of c-Myc during first 5-days of culture (dashed line), but the protein was detected on 7d in colonies consisting of 50–60 cells. (B) Immunofluorescence staining of Stat-3 (green). Stat-3 was not detected in 1d of PGCs culture (dashed line), but became detectable at 4d of culture mostly in the cytoplasm (dashed line), although we also observed small colonies of cells in which Stat-3 was nuclear (white line). Merged images are shown with DNA stained with Toto-3 (blue). Scale bar, 30 µm in B–E and G, scale bar 60 µm in F and H. (C) Diagram depicts LIF requirement in the reprogramming process. LIF was added to PGC after 1-, 2- or 4-days of culture. PGCs cultured in SCF, LIF and FGF-2 were used as control. After 10 days all cultures were stained for TNAP activity and the number of colonies was counted. We observed similar number of colonies in control and cultures in which LIF was added after 1-day (+++). Other cultures generated only one colony when LIF was added after 2-days, and no colonies formed when LIF added after 4-days.

Previous studies have shown that *c-Myc* is another known target of Blimp1 [11]. We know that *c-Myc* is expressed in EG cells but not in the PGC, as judged by PCR analysis, and by immunostaining using specific antibodies on PGCs isolated from E8.5 or 11.5 embryos, but it is detected in the nuclei of EG cells (Figure S3). When we analysed expression of c-Myc in PGCs during derivation of EG cells in the presence of FGF-2, LIF and SCF, we found a strong signal in PGCs forming large colonies consisting of 50–60 cells after 7 days of culture (Figure 2A). But we did not detect c-Myc during the first 6 days of culture (Figure 2A), nor in the older cultures where we observed many small colonies (data not shown). When EG-like colonies of 200–300 cells were formed after 10 days of culture, c-Myc was present in all the cells within these colonies (Figure S3). It should also be noted that c-Myc is a direct target of Stat-3, which in turn responds to LIF signaling (see below). This together with the loss of Blimp1 in cultured PGCs provides appropriate condition for its expression in EG-like cells.

As noted above, we also found that *Klf4* is differentially expressed in EG cells but not in PGCs, since we could not detect any transcripts or protein in E8.5 PGCs (Table 1, Figure S4A and S4B). We therefore examined expression of Klf-4 in cultured PGCs and found that PGCs after 1-day of culture did not express Klf-4 but after 3-days of culture we observed low levels of Klf-4 in all the cells we examined, and we detected a strong signal in large colonies (Figure S4 C). Notably, we now have evidence to show that Klf-4 is also a direct target of Blimp1 (Figure S5). Thus, the up-regulation of Dhx38, c-Myc and Klf-4 is consistent with the loss of Blimp1 in cultured PGCs.

### Activation of LIF/Stat3 signalling is required after 1 day of PGC culture

Next, we went on to examine changes in the signal transducer and activator of transcription-3 (Stat-3). We did not detect Stat-3 protein in PGCs from E8.5 embryos but we detected Stat-3 protein in late PGCs obtained from E12.5–14.5 gonads (Figure S6). We also detected Stat-3 in the cytoplasm and nuclei of EG cells (Figure 2 B). During the culture of E8.5 PGCs, we did not observe Stat-3 after 1-day culture but it was detectable after 4-days culture, when it localised predominantly in the cytoplasm of PGCs, except in a few cases where we observed a strong nuclear Stat-3 staining in PGCs that were forming small colonies (Figure 2 B). In older cultures, when larger PGC colonies were established, we could detect Stat-3 in both the cytoplasm and nuclei of these cells suggesting that there is transient localisation of Stat-3 in the nucleus.

Stat-3 is expressed in both ES and EG cells and the signalling pathway is activated by LIF [12]. The effect of LIF on PGCs is to enhance their proliferation and survival in vitro [13]. To gain further insight into the significance of LIF/Stat-3 signaling in the reprogramming process, we added LIF to PGCs cultures (SCF and FGF-2 were always present) starting at different time points, which we did on day 1, or after day 2 or day 4 of culture, instead of continuously for 10 days as we did previously (Figure 2C). We found that cultures with LIF added after 1-day of culture gave rise to similar numbers of EG-like colonies to those detected in controls. However, the addition of LIF after 2 days gave rise to only one colony, while the delay in the addition of LIF to the cultures after 4 days produced no EG-like colonies. Furthermore, when we abrogated the Jak/Stat-3 signalling pathway with the addition of 1 or 5 µM specific chemical inhibitor- WHI-P131, the formation of EG-like colonies was abolished, whereas control cultures treated with the vehicle only (DMSO) gave rise to many EG-like colonies. This result indicates that LIF is not essential for the first 24 hours, unlike FGF-2, which by contrast is essential only during the first 24 h of culture.

### Trichostatin A, an inhibitor of histone deacetylase can substitute for FGF-2

**Figure 3:**
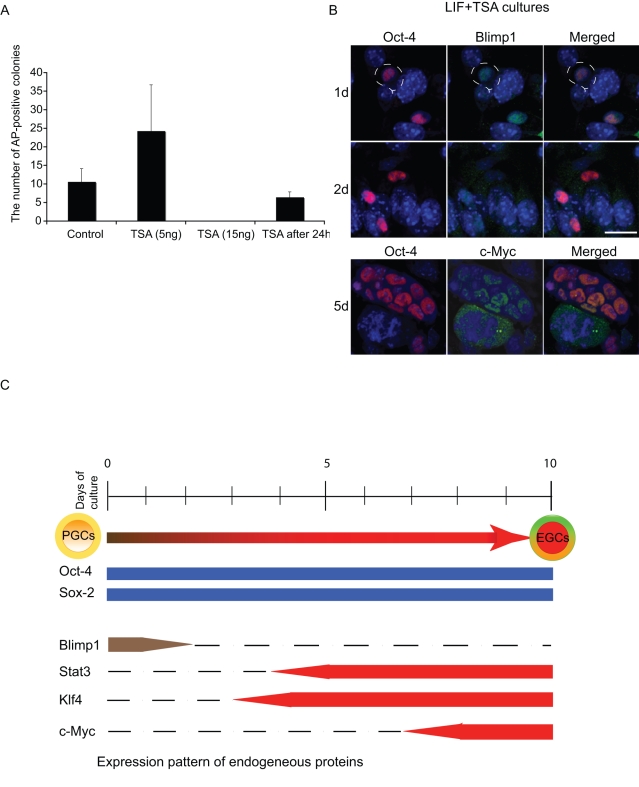
TSA replaces FGF-2 in the reprogramming process. (A) Approximately 80 PGCs from E 8.5 embryos were cultured in the presence of SCF, LIF and treated with TSA, at 5 or 15 ng/ml. In some cultures TSA (5 ng/ml) was added after 24-hours. PGCs cultured in SCF, LIF and FGF-2 were used as a control. After 10 days, cells were stained for TNAP activity and the number of colonies was counted. Graph shows combined results from three independent experiments for each TSA concentration. Error bars indicate standard deviation. Cultures treated with TSA (5 ng/ml) were reprogrammed more efficiently than PGCs in the control group, or cultures treated with TSA 24 hours later. TSA at 15 ng/ml was toxic to cells. (B) Immunofluorescence of TSA-treated cultures. Blimp1 (green) was down-regulated more rapidly compared to the control on 1d and was undetectable in 2d cultures. c-Myc (green) was detected in small colonies 5d cultures indicating an acceleration of the reprogramming process. Merged images are shown with DNA stained with Toto-3 (blue). Scale bar, 30 µm. (C) Model illustrating key events in the PGCs reprogramming to EG cells in the presence of FGF-2. Coloured boxes depict expression pattern of studied proteins. On day 1, cultured PGCs are in a repressive state due to expression of Blimp1/Prmt5 complex in nuclei of all PGCs. On day 2, PGC are released from lineage repression through the loss of Blimp1 (a brown box) and undergo further phenotypic changes leading to pluripotency when targets of Blimp1 such as Dhx38, c-Myc and Klf-4 are upregulated. On day 4 Stat-3 (a red box) translocates from cytoplasm-to-nuclei. Klf-4 is detected at low levels. On day 7 some cultured PGCs form large colonies. Cells of these colonies express c-Myc, while Prmt5 translocates from nuclei to the cytoplasm. Expression of Klf-4 and Dhx38 is at high levels in nascent EG cells. PGCs express Oct-4 and Sox2 throughout the culture period. The process is more efficient and is accelerated by 1–2 days when FGF-2 is replaced with TSA.

We decided to investigate if attempts at direct alteration of the epigenetic status of PGCs could substitute for the requirement of FGF-2. We investigated the impact of Trichostatin A (TSA), an inhibitor of histone deacetylases (HDACs), to see if it can trigger PGC dedifferentiation into EG cells in the absence of FGF-2. PGCs were cultured in the presence of TSA and LIF, but without FGF-2 for 3-days, followed by culture in medium with LIF only. While TSA at a concentration of 15 ng/ml was toxic, we found a striking effect following the addition of only 5 ng/ml of TSA, which resulted in the formation of more EG-like colonies when compared to the controls (LIF plus FGF-2)(Figure 3A), which indeed subsequently generated EG cells (Figure S7). We found that PGCs were responsive to TSA even when it was added on day 2 of culture, although there was a decline in the efficiency of generation of EG-like colonies from PGCs. Notably, performing these experiments with TSA, we also noticed that the EG-like colonies appeared 1–2 days earlier than in control cultures with LIF and FGF-2. Furthermore, we also found that in the down regulation of Blimp1 in the presence of TSA already occurred in PGCs after 1 day, while the expression of c-Myc was detected much earlier at 5 days after culture of PGCs (Figure 3B). These experiments demonstrate that TSA not only replaces FGF-2 signalling but it also accelerates dedifferentiation of PGCs into EG cells.

## Discussion

Here we have demonstrated the key sequence of events associated with reprogramming of PGCs to EG cells (Fig. 3C). Whereas PGCs show expression of many pluripotency genes, they are lineage restricted and cannot contribute to chimeras, while EG cells, which are their *in vitro* derivatives, can do so [5]. Our results suggest that a release from the constraints that maintain PGCs may be prerequisite. This follows with the down regulation of Blimp1 repressive complex that apparently has a role in the maintenance of early germ cell lineage. This is supported by our observation that Blimp1 was not down-regulated in PGCs cultured in the presence of LIF and SCF alone, which are conditions under which PGCs do not dedifferentiate to EG cells. Down-regulation of Blimp1 occurred only when either FGF-2 or TSA was present in the medium. The process of Blimp1 down-regulation and the derivation of EG cells were significantly accelerated when FGF-2 was substituted by TSA. A possible alternative approach to examine the role of Blimp1 in reprogramming of PGCs is to use Blimp1-deficient PGCs to generate EG cells, but unfortunately, this is not feasible since PGCs in E8.5 Blimp1-deficient mutant embryos only produce aberrant PGCs, which cease to proliferate altogether [1]. In contrast, Prmt5, which forms a complex with Blimp1, was observed in PGCs throughout the culture period. However, after the formation of large colonies resembling EG cells, we found that Prmt5 had translocated from nuclei-to-cytoplasm. This may be important for generating EG cells from PGCs, since our preliminary studies suggest that Prmt5 has a critical role in pluripotency, and for the derivation and maintenance of pluripotent stem cells. We are currently examining this role of Prmt5 in pluripotency (W. W. Tee et al., unpublished). The up-regulation of c-Myc at the time of initiation of self-renewal of EG cells may depend on LIF/Stat-3 signaling pathway following phosphorylation of Stat-3. Down-regulation of Blimp1 would also result in the transcriptional activation of target genes including c-Myc, Dhx38 and Klf-4, which are the targets of Blimp1 complex.

In our previous study we found that the presence of FGF-2 is critical during the first day but it is dispensable thereafter, when presumably the PGCs begin to switch from their *in vivo* developmental programme towards the formation of EG cells *in vitro* [5]. This study emphasises that the key events must occur within the first 24 hours. Further detailed studies are needed to investigate the early events associated with reprogramming of PGCs. In contrast, we found that LIF is not required until day 2 of culture, presumably this is when the LIF/Stat-3 signalling pathway assumes an important role, together with the survival factor, SCF. At present, the initial role of FGF-2 (or TSA) appears to be to trigger the down-regulation of repressor(s) such as Blimp1 in PGCs, which is then followed by a cascade of other events. Signalling molecules, such as FGF-2, can regulate access of transcription factors to genomic promoters by local chromatin modifications and trigger transcriptional and epigenetic changes that lead to EG cell phenotype. Notably, we also found that TSA, an HDAC inhibitor obviates the need for FGF-2. Moreover TSA accelerated and increased the efficiency of reprogramming process probably by making histone residues more acetylated and therefore the chromatin accessible to transcription factors that may promote more efficient progression from PGCs to pluripotent EG cells. Furthermore, Blimp1 has been shown to recruit histone deacetylase (HDAC) to repress targets, such *c-Myc*, which would be abrogated by TSA [14]. This may in part explain why TSA is effective in inducing reprogramming of PGCs to EG. Further experiments should reveal additional targets of the Blimp1 complex, some of which may play an important role during dedifferentiation of PGCs into EG cells.

Recent studies have shown that fetal and adult fibroblast cells can be reprogrammed into iPS cells by the introduction of four transcription factors, Oct-4, Sox-2, Klf-4 and c-Myc [15]–[18]. PGCs already show expression of Sox-2 and Oct-4, while c-Myc and Klf-4 are up-regulated during reprogramming to EG cells. Recent report on Klf family (Klf-2, Klf-4 and Klf-5) showed that these genes are important for the self-renewal of ES cells by regulating key pluripotency genes, including Nanog and their simultaneous deletion leads to differentiation of ES cells [19]. Thus, up-regulation of Klf-4 in 4-day cultured PGCs is an important event during dedifferentiation of PGCs into pluripotent stem cells. The efficiency of PGCs reprogramming is higher than reprogramming of somatic cells, while reprogramming of fibroblasts into pluripotent stem cell is also a gradual process that takes over 12–14 days of culture, and this is accompanied by epigenetic changes [20], [21].

We propose that reprogramming of PGCs to EG cells provides a tractable model system to discover other key events and factors, including epigenetic regulators, involved in this dedifferentiation event. This analysis may be particularly important for the discovery of the earliest events associated with the reprogramming events, which could deepen our understanding of the mechanism of reprogramming of PGCs as well as of somatic cells into pluripotent stem cells.

## Materials and Methods

### Mice and cell lines

Animals were treated according to guidelines approved by the Cambridge University Ethical Review Committee. MF1 females mated with homozygous 129 males were used to provide fetuses. The day of the vaginal plug was designated as embryonic day E 0.5. E11.5 PGCs were sorted by using magnetic beads preformed as described [22]. EG cell lines, 8.5 EGC-1 and 4-3 Rosa, were derived from E8.5 and −11.5 PGCs, respectively.

### cDNA synthesis and PCR

Single-cell cDNA libraries were generated as previously described [23]. cDNAs made from purified E11.5 PGCs and EG cells were described previously [24]. Sequences of PCR primers used in this study will be sent upon a request.

### Culture conditions

Dissection of PGC-containing tissues from E8.5 embryos, the culture conditions for derivation of EG cell lines and TNAP staining were as described [22]. For pulse-treated cultures, LIF at concentration 1200 IU/ml was added to culture media supplemented with FGF-2 (Invitrogene, 25 ng/ml) after 1, 2 or 4 days. After 10 days, cultures were stained for TNAP activity and EG-like colonies were counted. Stat-3 inhibitor, WHI-P131 (Sigma), was dissolved in DMSO (Sigma) as 1 mM stock solution and stored at −20°C. PGCs were cultured in WHI-P131 containing media at 1 or 5 µM concentration. As a control we treated cultures with an equal volume of the vehicle DMSO. For culture treated with Trichostatin A (TSA), PGCs were cultured with LIF and TSA. TSA (Sigma) was dissolved in at 1 mg/ml in ethanol and stored at −20°C.

### Immunofluorescence

Immunofluorescence of PGCs, EG cells or cultured PGCs in Lab-tek chambers (Nunc) was carried out as described [22]. Briefly, cells were fixed with 4% paraformaldehyde (PFA), washed three times with phosphate-buffered saline (PBS) permeabilized (PBS; 0.1% Triton X-100; 1 mg/ml bovine serum albumin). Primary antibody incubation was carried out overnight at 4°C, followed by three washes in PBS and incubation with secondary antibodies (Alexa 564, Alexa 488; Molecular Probes, Eugene) for 1 h at room temperature, and washed in PBS. Nuclei were counterstained with Toto-3 (Molecular Probes) and then mounted in vectashields (Vector Laboratories, Burlingame, CA). Cryosections of male or female gonads were carried out as described [24]. The following antibodies and dilutions were used: Oct-4 (BD Transduction Laboratories, San Diego, CA; 1∶250), TG1 (mouse germ-cell specific anti-SSEA1 monoclonal antibody; 1∶1), Blimp1 (rabbit polyclonal, Tooze lab, 1∶100) Prmt5 (Upstate, Charlottesville, VA; 1∶250), Dhx38 (Proteintech Group, Chicago, IL; 1∶200), Stat-3 (C-20, sc-482 1∶100), c-Myc (N-265, sc-764, 1∶200), Sox-2 (Y-17, sc-17320, 1∶100), GKLF (H-180, sc-20691, 1∶100) all from Santa Cruz Biotechnology, Inc.

## Supporting Information

Figure S1 Expression of Oct-4 and Sox-2 in cultured PGCs. 8.5 dpc PGCs were cultured in LIF and FGF-2 containing media on feeder cells expressing SCF. Immunostaining of Oct-4 (green) and Sox-2 (red) were performed on 1-, 3- or 7-days cultured PGCs. Cultured PGCs co-expressed Oct-4 and Sox-2 at high levels. Merged images are shown with DNA stained with Toto-3 (blue). Scale bar, 30 µm.
(2.08 MB EPS)

Figure S2 Expression of Blimp1/Prmt5 and Dxh38. 8.5 dpc PGCs and EG cells were stained with Blimp1 or Prmt5 specific antibody. (A) Blimp1 (green) is detected in nuclei of PGCs (dashed line) and not detected in EG cells. (B) Prmt5 (green) is detected in nuclei of 8.5 dpc PGCs but in EG cells is predominantly detected in their cytoplasm. (C) Cultured PGCs for different period of time were stained with Dxh38 antibody. Dhx38 (green) was not detected in 4-d cultured PGCs but strong signal was detected in somatic cells. After 7–8 d of culture PGCs Dhx38 was detected at low levels in small colonies and at high levels in large colonies. PGCs and EG cells were identified by using Oct-4 antibody (red). Merged images are shown with DNA stained with Toto-3 (blue). Scale bar 50 µm.
(2.69 MB EPS)

Figure S3 c-Myc expression. (A) RT-PCR analysis was performed on 11.5 dpc PGCs (purity 85%) and EG cells. PGCs did not express c-Myc. In contrast EG cells expressed c-Myc at high level. (B–D) Immunofluorescence staining of c-Myc (green) in PGCs and EG cells. PGCs and EG cells were identified by using Oct-4 antibody (red). (B–C) 8.5 and 11.5 dpc PGCs, respectively, did not express c-Myc. Few somatic cell surrounding 8.5 dpc PGC expressed c-Myc (arrowhead) (D) EG cells expressed c-Myc at high levels. Merged images are shown with DNA stained with Toto-3 (blue). Scale bar 50 µm.
(3.11 MB EPS)

Figure S4 Expression of Klf-4. Immunofluorescence staining of Klf-4 (green) was performed on (A–B) 8.5 dpc PGCs and EG cells and © cultured PGCs for different period of time. PGCs and EG cells were co-stained with antibodies against Oct-4 (red). (A) 8.5 dpc PGCs did not express Klf-4 (B) EG cells expressed Klf-4 at high levels. (C) 1-day cultured PGCs did not express Klf-4, but after 3-days of culture we detected a week signal, which was stronger in PGCs forming large colonies after 8-days of culture. Merged images are shown with DNA stained with Toto-3 (blue). Scale bar, 30 µm.
(2.45 MB EPS)

Figure S5 Klf-4 is Blimp1 target. Association of BLIMP1 with the KLF4 promoter region in vivo. (A) Position of putative BLIMP1 binding site −384 to −374 upstream of the KLF4 transcription start (TS) site. (B) Chromatin prepared from BLIMP1 expressing H929 or U266 myeloma cells or non-expressing Daudi B-cells was immunoprecipitated with control rabbit IgG or anti-BLIMP1 and the amount of KLF4 sequence present in each quantified by real-time PCR. Data is displayed as enrichment relative to control IgG and plotted using the position of the forward primer. Note: the position of the binding site refers to the sequence AGAAGAAAGGG.
(0.74 MB EPS)

Figure S6 Expression of Stat-3 in 12. 5 to 14.5 dpc germ cells, and EG cells. Cryosections of genital ridges isolated from 12.5, 13.5 and 14.5 old female and male embryos were stained with Stat-3 antibody. Stat-3 (green) was detected in cytoplasm of germ cells in both males and females. Germ cells were identified with Oct-4 antibody (red). At 14.5 dpc male and female germ cells we did not detect Oct-4. In EG cells Stat-3 was detected in nuclei and cytoplasm. Merged images are shown with DNA stained with Toto-3 (blue). Scale bar 50 µm.
(3.21 MB EPS)

Figure S7 TSA can replace FGF-2 to generate EG cells. EG cell lines generated in the presence of TSA resembled those obtained in the presence of FGF-2. (A) Undifferentiated EG cells were stained with several antibodies used for the characterisation of pluripotent stem cells. EG cells expressed SSEA-1 (red), EMA-1 (red), Oct-4 (red) and Sox-2 (green). (B) Differentiation potential of EG cells was studied via embryoid body formation. Cells differentiated into several lineages as judged by the expression of collagen IV (green), GDNF (red) and fibronectin (green). Merged images are shown with DNA stained with Toto-3 (blue). Scale bar 50 µm.
(1.36 MB EPS)

## References

[1] Ohinata Y, Payer B, O'Carroll D, Ancelin K, Ono Y (2005). Blimp1 is a critical determinant of the germ cell lineage in mice.. Nature.

[2] Ancelin K, Lange UC, Hajkova P, Schneider R, Bannister AJ (2006). Blimp1 associates with Prmt5 and directs histone arginine methylation in mouse germ cells.. Nat Cell Biol.

[3] Matsui Y, Zsebo K, Hogan BLM (1992). Derivation of pluripotent embryonic stem cells from murine primordial germ cells.. Cell.

[4] Resnick JL, Bixler LS, Cheng L, Donovan PJ (1992). Long-term proliferation of mouse primordial germ cells in culture.. Nature.

[5] Durcova-Hills G, Adams IR, Barton SC, Surani MA, McLaren A (2006). The role of exogenous fibroblast growth factor-2 on the reprogramming of primordial germ cells into pluripotent stem cells.. Stem Cells.

[6] Hajkova P, Erhardt S, Lane N, Haaf T, El-Maarri O (2002). Epigenetic reprogramming in mouse primordial germ cells.. Mech Dev.

[7] Youngren KK, Coveney D, Peng X, Bhattacharya C, Schmidt LS (2005). The Ter mutation in the dead end gene causes germ cell loss and testicular germ cell tumours.. Nature.

[8] Muller AJ, Teresky AK, Levine AJ (2000). A male germ cell tumor-susceptibility-determining locus, pgct1, identified on murine chromosome 13.. Proc Natl Acad Sci U S A.

[9] Kimura T, Suzuki A, Fujita Y, Yomogida K, Lomeli H (2003). Conditional loss of PTEN leads to testicular teratoma and enhances embryonic germ cell production.. Development.

[10] Kimura T, Tomooka M, Yamano N, Murayama K, Matoba S (2008). AKT signaling promotes derivation of embryonic germ cells from primordial germ cells.. Development.

[11] Lin Y, Wong K, Calame K (1997). Repression of *c-myc* transcription by Blimp-1, an inducer of terminal B cell differentiation.. Science.

[12] Niwa H (2007). How is pluripotency determined and maintained?. Development.

[13] Cheng L, Gearing DP, White LS, Compton DL, Schooley K (1994). Role of leukemia inhibitory factor and its receptor in mouse primordial germ cell growth.. Development.

[14] Yu J, Angelin-Duclos C, Greenwood J, Calame K (2000). Transcriptional repression by blimp-1 (PRDI-BF1) involves recruitment of histone deacetylase.. Mol Cell Biol.

[15] Takahashi K, Yamanaka S (2006). Induction of pluripotent stem cells from mouse embryonic and adult fibroblast cultures by defined factors.. Cell.

[16] Okita K, Ichisaka T, Yamanaka S (2007). Generation of germline-competent induced pluripotent stem cells.. Nature.

[17] Wernig M, Meissner A, Foreman R, Brambrink T, Ku M (2007). In vitro reprogramming of fibroblasts into a pluripotent ES cell-like state.. Nature.

[18] Maherali N, Sridharan R, Xie W, Utikal J, Eminli S (2007). Directly reprogrammed fibroblasts show global epigenetic remodelling and widespread tissue contribution.. Cell Stem Cell.

[19] Jiang J, Chan Y-S, Loh Y-H, Cai J, Tong G-Q (2008). A core Klf circuitry regulates self-renewal of embryonic stem cells.. Nat Cell Biol.

[20] Brambrink T, Foreman R, Welstead GG, Lengner CJ, Wernig M (2008). Sequential expression of pluripotency markers during reprogramming of mouse somatic cells.. Cell Stem Cell.

[21] Stadtfeld M, Maherali N, Breault DT, Hochedlinger K (2008). Defining molecular cornerstones during fibroblast to iPS cell reprogramming in mouse.. Cell Stem Cell.

[22] Durcova-Hills G, McLaren A, Lanza R, Gearhart J, Hogan B, Melton D, Pedersen R, Thomas J, West M (2004). Isolation and maintenance of murine embryonic germ cell lines.. Handbook of Stem Cells, Volume 1..

[23] Saitou M, Barton SC, Surani MA (2002). A molecular programme for the specification of germ cell fate in mice.. Nature.

[24] Adams IR, McLaren A (2004). Identification and characterisation of mRif1: A mouse telomere-associated protein highly expressed in germ cells and embryo-derived pluripotent stem cells.. Dev Dyn.

